# Cynthichlorine Extracted from Ascidian *Cynthia savignyi* from Djibouti: Optimization of Extraction, In Vitro Anticancer Profiling, and In Silico Approach

**DOI:** 10.3390/md23040172

**Published:** 2025-04-16

**Authors:** Fatouma Mohamed Abdoul-Latif, Houda Mohamed, Ibrahim Houmed Aboubaker, Omaima Saoudi, Ayoub Ainane, Ali Merito Ali, Stefano Cacciatore, Luiz Fernando Zerbini, Abdelmjid Abourriche, Tarik Ainane

**Affiliations:** 1 Medicinal Research Institute, Center for Research and Study of Djibouti, Djibouti P.O. Box 486, Djibouti; abdoulhouda@yahoo.fr (H.M.); ibrahimhoumed@yahoo.fr (I.H.A.); alimerito@hotmail.fr (A.M.A.); 2 Peltier Hospital of Djibouti, Djibouti P.O. Box 2123, Djibouti; 3 Superior School of Technology (EST-Khenifra), University of Sultan Moulay Slimane, BP 170, Khenifra 54000, Morocco; sao.omaima@gmail.com (O.S.); a.ainane@usms.ma (A.A.); 4 Bioinformatics Unit, International Centre for Genetic Engineering and Biotechnology (ICGEB), Cape Town 7925, South Africa; stefano.cacciatore@icgeb.org; 5 Cancer Genomics Group, International Centre for Genetic Engineering and Biotechnology (ICGEB), Cape Town 7925, South Africa; luiz.zerbini@icgeb.org; 6 Laboratory of Analytical and Molecular Chemistry, Faculty of Sciences Ben M’sik, University of Hassan II, Casablanca 20700, Morocco; a.abourriche@gmail.com

**Keywords:** ascidian, cancer, *Cynthia savignyi*, cynthichlorine, cytotoxicity, extraction, bioinformatics

## Abstract

This work focuses on the extraction of cynthichlorine from the ascidian *Cynthia savignyi*, a molecule that has potential promise as an anticancer agent. The main objective was to optimize the extraction conditions and evaluate the cytotoxic activity of cynthichlorine in tumor cell lines. Two extraction methods, maceration and Soxhlet extraction, were compared, with maceration showing a significantly higher yield (2.2 ± 0.2%) compared to Soxhlet extraction (1.0 ± 0.2%). An optimization of the factors influencing the extraction was performed using the Box–Behnken method, showing that the extraction temperature and time have a negative impact on the yield, with the optimal conditions of temperature being below 25 °C and those of extraction time being below 12 h. Cytotoxic activity assessment revealed the marked inhibition of cell growth in all tested lines (U87-MG, U2OS, NCI-N87, HCT116, and A2780), with IC_50_ values ranging from 0.162 µg/mL in U87-MG to 0.576 µg/mL in NCI-N87. Finally, computational analysis showed that cynthichlorine exhibits high electronic stability and notable affinity for some biological targets, including NM23-H2, suggesting its potential as a targeted therapy in cancer treatment. These results pave the way for future studies on the therapeutic use of cynthichlorine.

## 1. Introduction

**Figure 1:**
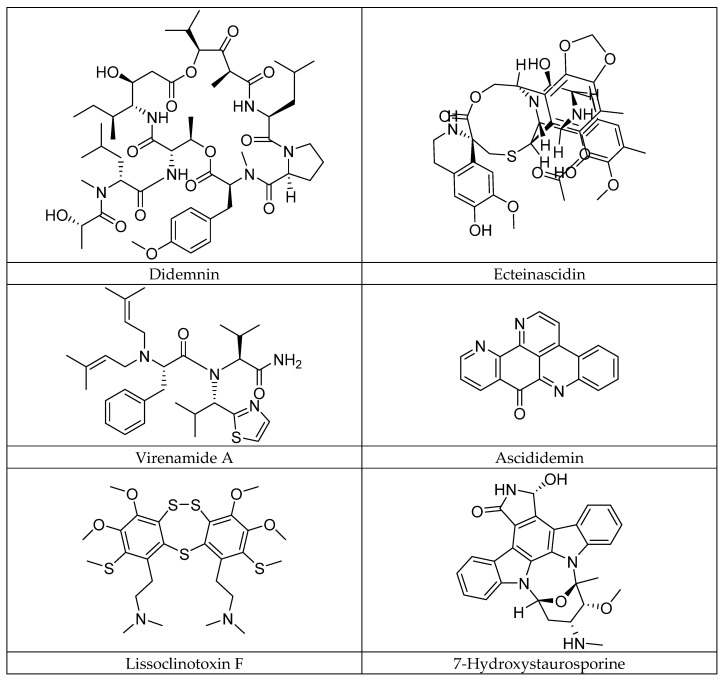
Some anticancer alkaloids of ascidian origin.

Marine ascidians, invertebrate organisms of the class Ascidiacea and phylum Chordata, play a fundamental role in the balance of marine ecosystems [1,2]. As biological filters, they intervene in the nutrient cycle and serve as a habitat for various marine species [3]. In addition, they are potential reservoirs of bioactive secondary metabolites [4]. These chemical compounds, produced by these marine organisms, mainly serve as defense mechanisms against predators and pathogens. Among these metabolites, marine alkaloids stand out for their exceptional pharmacological properties, including their antibiotic, cytotoxic, and anticancer effects [5]. To date, more than 300 ascidian alkaloids have been identified, several of which have shown remarkable biological activities, generating increasing interest in the field of marine pharmacology [6]. Marine alkaloids, although sharing some similarities with terrestrial alkaloids, are distinguished by their unique chemical structures and distinct biological mechanisms of action. These compounds possess a wide range of biological activities, such as the inhibition of topoisomerases and cyclin kinases, as well as immunosuppressive effects [7]. These activities are particularly relevant in the context of cancer treatment, where some marine alkaloids have shown remarkable anticancer and antitumor effects (Figure 1). Among these molecules, didemnin [8] and ecteinascidin [9], two well-regarded marine alkaloids, have been investigated for their efficacy in cancer treatment, where didemnin, in particular, has shown anticancer activity by inhibiting cell division and interfering with DNA structure, making it a promising candidate for therapies against different types of cancers. In addition, linear tripeptides such as virenamides A–C, extracted from the ascidian *Diplosoma virens*, have shown strong cytotoxic activity against P388 cells, with the notable inhibition of the enzyme topoisomerase II, a key marker in anticancer drug development [10]. Other compounds, such as ascididemine, an alkaloid derived from pyrido[2,3,4-kl]acridine, have demonstrated impressive cytotoxicity against human tumor cells, including P-388 leukemia and colorectal cancer [11]. Research on marine alkaloids is actively ongoing, with promising findings in the field of cancer. Compounds such as lissoclinotoxins E and F, extracted from the ascidian *Didemnum* harvested in the Philippines, have shown the marked inhibition of the growth of human mammary carcinoma cells, with particular efficacy against PTEN-deficient cell lines [12]. These results open the way for the use of these compounds in the treatment of cancers resistant to conventional therapies. Similarly, alkaloids from the ascidian *Aplidium conicum* have demonstrated significant cytotoxic activity against human cancer cells, including those of breast carcinoma and human leukemia [13]. Furthermore, staurosporine derivatives, such as 7-hydroxystaurosporine, are currently in clinical trials for their potential to inhibit the growth of T-cell lymphomas, illustrating the diversity of therapeutic applications of marine alkaloids in the treatment of different types of cancers [14].

**Figure 2:**
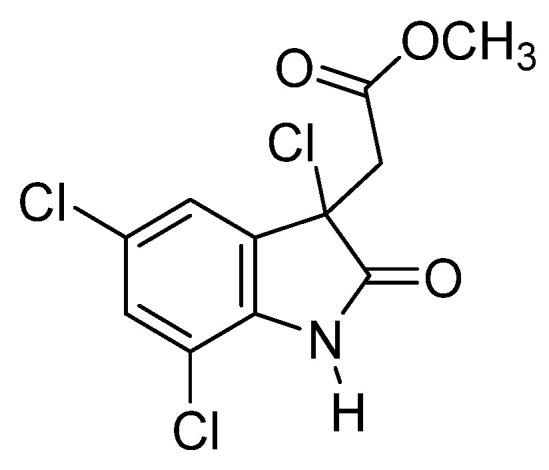
Structure of cynthichlorine.

A particularly notable ascidian species is *Cynthia savignyi*, namely due to its promising bioactive metabolites [15]. Found in many seas worldwide, this species represents a potential source of therapeutic compounds to treat various diseases. Among these metabolites, cynthichlorine (Figure 2), a trichlorinated alkaloid derived from oxine-dole, was isolated from ethereal extracts of the ascidian [16]. Cynthichlorine possesses several bioactive properties, including antifungal, antibacterial, and cytotoxic activity. It has been shown to inhibit the growth of several fungal pathogens, such as *Botrytis cinerea* and *Verticillium albo-atrum*, which are responsible for fungal diseases affecting plants [15]. Furthermore, cynthichlorine exhibits strong antibacterial activity against bacteria such as *Escherichia coli*, *Pseudomonas aeruginosa*, and *Agrobacterium radiobacter*, which are implicated in human and plant infections [16]. The cytotoxicity of cynthichlorine was assessed, and its median lethal dose (LD_50_) was determined to be 48.5 µg/mL, indicating moderate toxicity. This toxicity profile suggests that cynthichlorine could be used as a potential therapeutic agent, although further studies are needed to confirm its safety and efficacy in animal and human models [16].

However, cynthichlorine extraction remains a major challenge. Despite the use of various extraction techniques, optimizing yields while preserving the integrity of the bioactive compounds remains complex. It is in this context that the importance of this study becomes evident. The main objective of this research is to optimize methods for extracting cynthichlorine from *Cynthia savignyi*. This study will aim to compare different extraction techniques to evaluate their effectiveness in terms of the yield and preservation of the biological activity of cynthichlorine. In addition, in vitro tests will be performed to evaluate the anticancer activity of this compound, particularly on human cancer cell lines. Finally, an in silico analysis of cynthichlorine will be performed to model its interactions with specific biological targets and elucidate its potential mechanisms of action. This study is essential for the development of new anticancer therapies and for the optimization of the extraction of bioactive compounds of marine origin.

## 2. Result

### 2.1. Study of the Improvement of the Extraction Method

**Figure 3:**
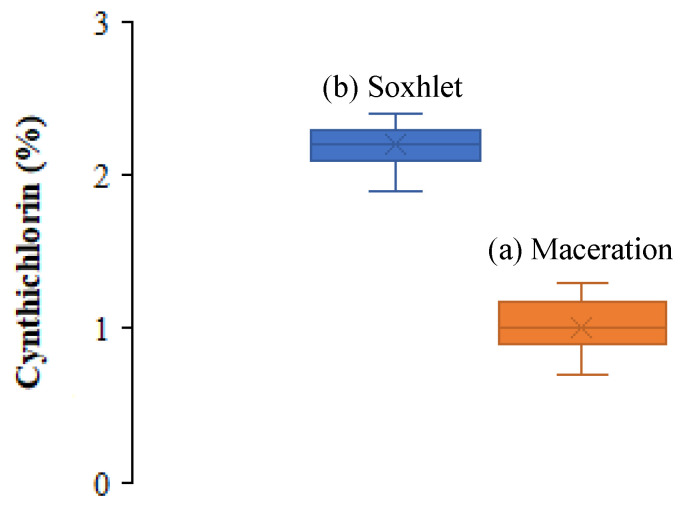
Boxplot illustrating the distribution of cynthichlorine yields of (**a**) maceration and (**b**) Soxhlet extraction methods.

In the framework of the evaluation of the yield of cynthichlorine extracted from the ascidian *Cynthia savignyi*, two extraction methods were tested: maceration and Soxhlet extraction. After several tests, the yields obtained were compared and statistically analyzed using Student’s *t*-test, and the results are summarized in Table 1. The average yields obtained showed a clear difference between the two methods, with an average of 2.2 ± 0.2% for maceration against 1.0 ± 0.2% for Soxhlet. The analysis of the results revealed a significant difference between the two processes, confirmed by the applied statistical test and according to the boxplot graphic representation (Figure 3). Maceration allowed a significantly higher yield than Soxhlet extraction; this variation can be attributed to the thermal sensitivity of cynthichlorine, thus explaining the decrease in yield observed with the Soxhlet method, which relies on high temperature extraction. The increase in temperature could alter or partially degrade the molecule, thus reducing its extraction efficiency. Consequently, maceration is recommended as the optimal extraction method; preserving the integrity of cynthichlorine, it allows the obtaining of a higher yield. These results demonstrate that, under the experimental conditions applied, maceration represents a more suitable and efficient approach for the extraction of cynthichlorine [17].

### 2.2. Optimization of Factors Influencing Extraction by Maceration

After obtaining preliminary results confirming that the yield of cynthichlorine favors maceration extraction, an optimization study of the factors influencing this process was conducted by applying the Box–Behnken experimental method with a single center [18]. This approach made it possible to evaluate the influence of temperature, extraction time, and alcohol concentration on the extraction yield. The levels studied for each factor are presented in Table 2. An experimental plan was designed to test different combinations of these factors and measure the yields obtained. The results of the 13 tests, modeled by the adjusted statistical model, are summarized in Table 3.

The fitting of the linear polynomial model to the experimental results allowed us to estimate the coefficients of the studied factors, as shown in Table 4. The analysis of the experimental results highlighted notable variations in the yield depending on the conditions applied. A mean value of 2.2% was observed with a standard deviation of 0.2. The maximum yield of 3.1% was obtained under optimal conditions, specifically when the temperature was 25 °C, the reaction time was 12 h, and the alcohol was 75%. In contrast, the lowest yield, 1.7%, was measured under suboptimal conditions, defined by a temperature of 35 °C, a reaction time of 24 h, and an alcohol percentage of 75%. The analysis of the estimated coefficients showed that the temperature and time had a significant negative effect on the yield, with respective coefficients of −0.3 and −0.2. On the other hand, the alcohol concentration presented a lower coefficient of −0.1, which suggests that its effect on the extraction yield is not very significant. In addition, the interactions between the factors showed low values, close to zero, indicating that they did not have a notable impact on the yield.

Analysis of Variance (ANOVA) was performed to assess the significance of the effects of the factors. The results are summarized in Table 5. It was observed that the temperature (*p* = 0.01) and time (*p* = 0.03) had a statistically significant effect on the extraction yield, while the alcohol concentration (*p* = 0.56) did not show a significant effect.

The evaluation of the model fit statistics (Table 6) revealed a coefficient of determination (R^2^ = 0.78), reflecting a good correlation between the factors studied and the yield obtained. However, the adjusted R^2^ = 0.56 suggests that some non-significant variables could be excluded to improve the quality of the model [19].

**Figure 4:**
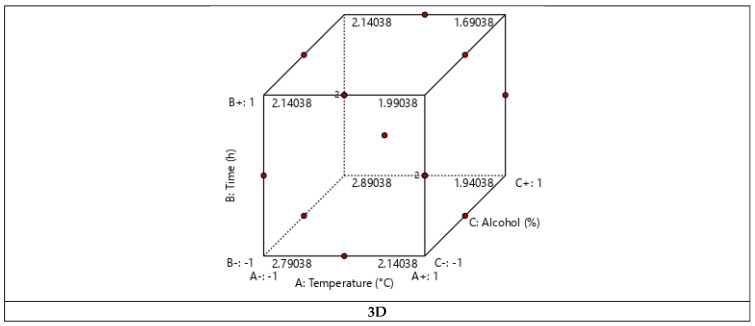

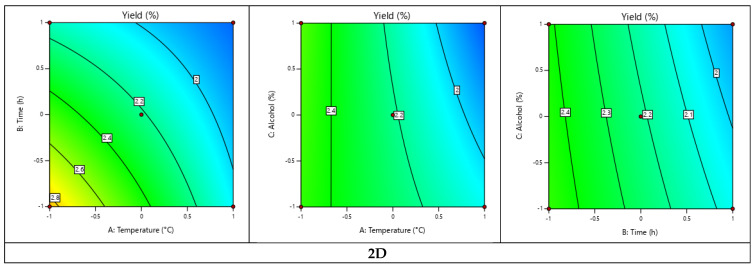
Three-dimensional and two-dimensional graphical representations of the experimental design of the cynthichlorine yield.

Three-dimensional and two-dimensional graphical representations were made to visualize the evolution of the yield according to the three factors studied (Figure 4). These representations confirmed that the temperature and the extraction time have a negative effect on the yield, thus proving that cynthichlorine is sensitive to heat and that a low temperature extraction process (below 25 °C) and short extraction time (less than 12 h) are more favorable, providing more remarkable yields.

### 2.3. Evaluation of Cytotoxic Activities

The cytotoxic activity of cynthichlorine and other reference anticancer agents was assessed using the CellTiter-Glo assay, a luminescent method for quantifying cell viability by measuring intracellular ATP, an indicator of active tumor cell metabolism. This study was conducted on five distinct cancer cell lines: U87-MG (glioblastoma), U2OS (osteosarcoma), NCI-N87 (gastric adenocarcinoma), HCT116 (colorectal carcinoma), and A2780 (serous ovarian carcinoma). The results presented in Table 7 allowed for the determination of the median inhibitory concentration (IC_50_), a key parameter that indicates the effectiveness of each compound in blocking the growth of tumor cells.

The analysis of the cytotoxic activity of cynthichlorine demonstrated the significant inhibition of cell growth across all lines tested. The IC_50_ values recorded ranged from 0.162 µg/mL for U87-MG to 0.576 µg/mL for NCI-N87, indicating variable sensitivity across tumor lines. The most marked inhibition was observed on the U87-MG line, suggesting notable efficacy against glioblastoma. Conversely, the most moderate response was observed on NCI-N87, revealing the lower sensitivity of this line to cynthichlorine.

Comparisons with other chemotherapeutic agents allowed for the assessment of differences in cytotoxicity across cell lines. Cisplatin showed particularly high activity against U2OS and NCI-N87, with extremely low IC_50_ values ranging from 0.002 to 0.004 µg/mL. In contrast, its efficacy was more limited against A2780, where cynthichlorine showed competitive activity with an IC_50_ of 0.529 µg/mL. For U87-MG, cynthichlorine displayed slightly higher cytotoxicity than cisplatin. Temozolomide, the standard agent for the treatment of glioblastoma, demonstrated particularly high efficacy against U87-MG, with an IC_50_ of 0.003 µg/mL, much lower than that of cynthichlorine. However, its activity was more restricted to other cell lines, with IC_50_ values exceeding 0.7 µg/mL, whereas cynthichlorine maintained more marked cytotoxicity, notably on U2OS, NCI-N87, HCT116, and A2780.

The evaluation of oxaliplatin revealed a particularly high cytotoxicity against HCT116, with an IC_50_ of 0.002 µg/mL, significantly lower than that of cynthichlorine (IC_50_ = 0.505 µg/mL). On the other lines, its efficacy was more moderate, often comparable to that of cynthichlorine. However, the latter demonstrated better cytotoxicity against U87-MG, with an IC_50_ of 0.162 µg/mL versus 0.387 µg/mL for oxaliplatin. Paclitaxel, on the other hand, exerted strong activity against A2780, with an IC_50_ of 0.003 µg/mL, significantly lower than that of cynthichlorine. A comparable or even slightly higher efficacy than cynthichlorine was noted against U87-MG and HCT116, while lower cytotoxicity was observed on U2OS and NCI-N87.

The overall analysis of these results identified several key aspects regarding the cytotoxic activity of cynthichlorine. It demonstrated moderate to high efficacy against all cell lines tested, distinguishing itself from temozolomide, whose activity mainly targeted U87-MG. Its activity against glioblastoma was particularly promising, outperforming cisplatin and oxaliplatin in this cell line. However, cynthichlorine was not the most potent agent against A2780 and HCT116, where paclitaxel and oxaliplatin showed superior activity.

These observations confirm the promising potential of cynthichlorine as an anticancer cytotoxic agent, particularly for glioblastoma and certain cell lines where it outperformed several conventional treatments [20].

### 2.4. Computational Analyses

#### 2.4.1. Bioinformatics of Quantum Descriptors of Cynthichlorine

**Figure 5:**
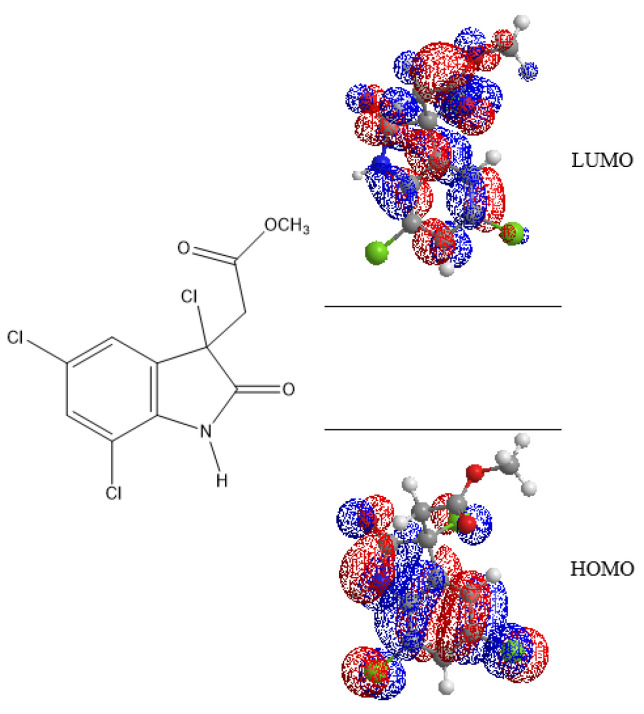
Energies LUMO and HOMO of cynthichlorine molecular orbitals.

The computational analysis of cynthichlorine was performed using the Merck Molecular Force Field 94 (MMFF94) method to model the interatomic interactions within the molecule in molecular mechanics. Seven (7) electronic descriptors were extracted to characterize its reactive properties, including the energies of the frontier molecular orbitals, occupied (E_HOMO_) and unoccupied (E_LUMO_), as well as the molecular gap energy (E_GAP_), a key parameter influencing electronic stability and chemical reactivity (Figure 5). Other quantum descriptors, such as chemical hardness, electronegativity, the electrophilicity index, and molecular flexibility, were calculated to obtain a more detailed characterization of the electronic and structural properties of the molecule (Table 8).

The results revealed that the E_HOMO_ was determined to be −9.441 eV, indicating that the molecule has a low propensity to donate electrons. This particularly negative value reflects increased stability and low oxidizability, suggesting that cynthichlorine is not a good electron donor. The calculated E_LUMO_ was −0.619 eV, reflecting a moderate capacity to accept electrons. This characteristic could influence its interactions with biological targets, in particular by limiting its ability to undergo reductions in a physiological environment [21].

E_GAP_ was estimated to be 8.822 eV, indicating high electronic stability and low chemical reactivity. Such an energy difference is generally associated with low redox activity, which can influence the bioavailability and the interaction with biological macromolecules. A molecule with a high gap is often more resistant to metabolic degradation and has a greater selectivity in its interactions, thus reducing the risk of unwanted side effects [22].

The η, determined at 4.411 eV, confirmed that cynthichlorine is not very reactive towards nucleophilic or electrophilic attacks. A molecule with a high hardness generally has a high chemical stability and a low tendency to undergo spontaneous electronic reactions. This stability could contribute to a prolonged half-life in a biological medium, which is an asset in pharmacokinetics [23]. The χ was evaluated at 5.030 eV, indicating an intermediate affinity for electrons. This property could influence the solubility of the molecule and its ability to form non-covalent interactions with biological receptors [24].

The ω, measured at 2.868 eV, allowed us to evaluate the ability of the molecule to interact with electron-rich biomolecules. A moderate value suggests that cynthichlorine could interact with some enzymes or proteins without being an aggressive alkylating agent. Too high electrophilicity is often associated with increased toxicity, which does not seem to be the case here [25]. In parallel, the S, estimated at 0.113 eV^−1^, revealed that the molecule presents a notable structural rigidity, which could influence its adaptation to various biological targets. A rigid molecule is often more selective in its interaction with well-defined active sites, which could play a key role in its mechanism of action [26].

The results obtained highlighted several fundamental aspects of cynthichlorine as a potential bioactive molecule. Its high energy gap and high chemical hardness suggest that it could be stable in a physiological environment, thus limiting its rapid metabolic degradation. This feature could prolong its therapeutic effect while reducing nonspecific interactions that could lead to side effects. In addition, its moderate electrophilicity index indicates potential selectivity towards certain biological targets without inducing excessive toxicity. However, its low molecular flexibility could restrict its interactions with certain types of receptors.

#### 2.4.2. Molecular Docking 

The molecular docking of cynthichlorine was achieved by targeting three key proteins involved in the regulation of transcription and chromatin structure, namely 8JUY (ATAD2), 3BBB (NM23-H2), and 8HPP (INTS3/SAGE1). These proteins play a fundamental role in transcriptional regulation mechanisms, influencing biological processes at the molecular level that have direct implications in cancer [27]. ATAD2, as an ATP-dependent histone chaperone, regulates the chromatin structure and the associated gene transcription [28]. NM23-H2 is involved in the transcriptional activation of the c-myc gene, a major oncogene, by modulating DNA structure and interacting with DNA secondary structures [29]. Finally, INTS3/SAGE1 regulates transcription by directly interacting with RNA polymerase II, a direct target in the transcription of genes involved in cancer [30]. These three proteins share transcriptional regulation mechanisms and represent potential targets for therapeutic strategies in cancer treatment.

**Figure 6:**
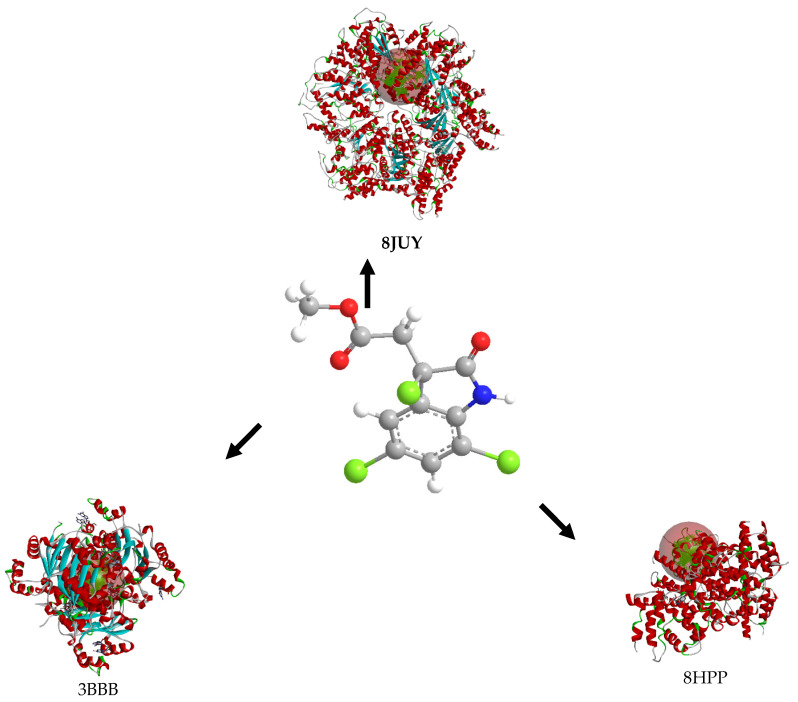
Three-dimensional structural interactions of complexes formed between cynthichlorine and proteins 8JUY, 3BBB, and 8HPP.

**Figure 7:**
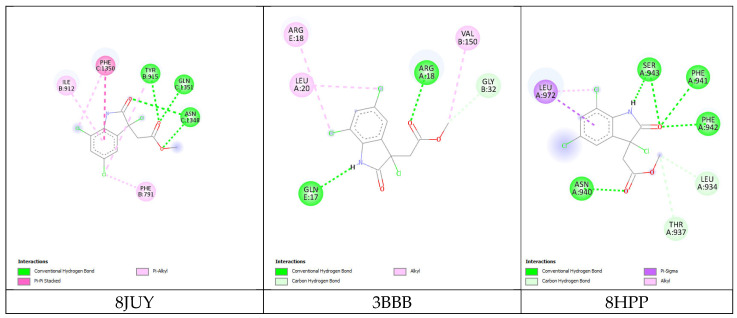
Two-dimensional structural interactions of complexes formed between cynthichlorine and proteins.

**Figure 8:**
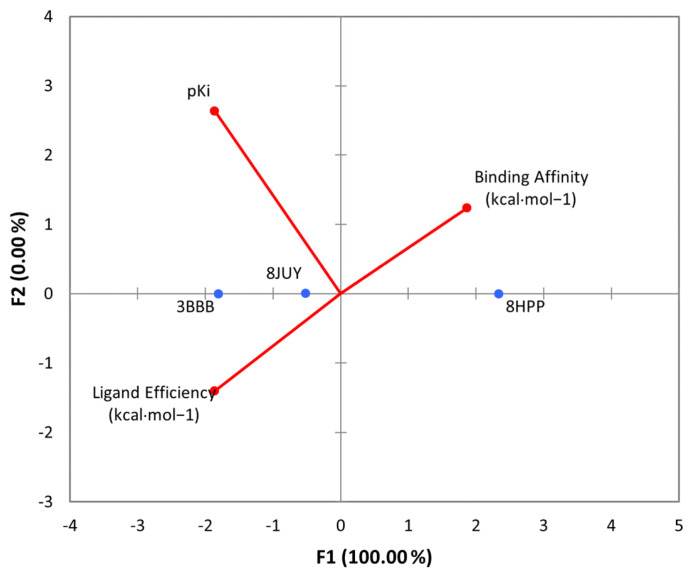
PCA of all parameters found for the molecular dockings of cynthichlorine and proteins 8JUY, 3BBB, and 8HPP.

The results of the 3D and 2D structural interactions formed between cynthichlorine and these proteins are illustrated in Figure 6 and Figure 7, while Table 9 presents the energetic data of the different complex dockings performed, thus Figure 8 gives the correlation between the parameters found for the three molecular dockings. Regarding the results obtained for each protein, for 8JUY (ATAD2), the binding affinity was measured at −6.5 kcal/mol, indicating a relatively stable interaction, but less strong than that observed for 3BBB (NM23-H2), whose binding affinity was calculated at −7.0 kcal/mol, the lowest free energy among the three proteins. This value is accompanied by a pKi of 5.13, which confirms a strong inhibition of cynthichlorine, and a binding efficiency of 0.3889 kcal/mol, highlighting the efficiency of the interaction between the ligand and the target. For 8HPP (INTS3/SAGE1), the results show the lowest binding affinity with −5.4 kcal/mol, accompanied by a pKi of 3.96 and a binding efficiency of 0.3000 kcal/mol, suggesting a less optimal interaction compared to the other two proteins [31].

In conclusion, NM23-H2 stands out as the most promising target for cynthichlorine, showing the best binding affinity, a high pKi, and the highest binding efficiency. ATAD2 is also an interesting target, although the interaction is slightly less strong. Finally, INTS3/SAGE1, with its less optimal results, might not be the preferred target for further studies. These results open perspectives for the development of cynthichlorine as a potential therapeutic agent, specifically targeting proteins involved in transcriptional regulation and cancer progression.

## 3. Discussion

The primary objective of this study was to optimize the extraction of cynthichlorine, a bioactive compound extracted from the Djibouti ascidian *Cynthia savignyi*, and to evaluate its anticancer potential in vitro. This compound, which has attracted increasing interest in pharmacological research [15,16], is being studied for its cytotoxic properties and its possible applications in the treatment of cancer, particularly glioblastoma. The study also aimed to explore the mechanism of action of cynthichlorine, in particular its chemical stability, as well as its interactions with target proteins linked to cancer progression. The secondary objectives of the study consisted of comparing different extraction methods, optimizing extraction conditions, and performing an in silico analysis to model the molecular interactions of cynthichlorine with specific protein targets.

The results of the study showed that the maceration method was significantly more efficient than Soxhlet extraction in terms of yield, with respective values of 2.2 ± 0.2% and 1.0 ± 0.2%. This difference was confirmed by statistical analysis, highlighting that maceration better preserves the integrity of cynthichlorine, particularly due to its thermal sensitivity. Soxhlet extraction showed a lower yield, possibly due to the thermal degradation of cynthichlorine at higher temperatures, a well-known phenomenon for thermosensitive bioactive compounds. The optimization of extraction conditions revealed that extraction temperature and time negatively affected the yield. The best conditions were obtained at a temperature below 25 °C and an extraction time of less than 12 h, thus confirming the thermal fragility of cynthichlorine and the need for low-temperature extraction methods to ensure optimal yield. Regarding anticancer activity, cynthichlorine showed the marked inhibition of cell growth against the U87-MG cell line, associated with glioblastoma, with an IC_50_ of 0.162 µg/mL, suggesting interesting therapeutic potential against this type of cancer. However, it did not outperform some chemotherapeutic agents against other cell lines, such as A2780 (ovarian adenocarcinoma) and HCT116 (colorectal cancer), indicating that its efficacy may be specific to certain cancer types. In silico analysis revealed that cynthichlorine has relatively high stability, with a high energy gap and low electron-donating capacity. These properties are advantageous for its bioavailability, potentially making it more stable in the body. Molecular docking simulations showed stable interactions with ATAD2, NM23-H2, and INTS3/SAGE1 proteins, with the best affinity observed with NM23-H2. These results suggest that cynthichlorine may have a mechanism of action modulating the activity of these proteins, which are involved in the cellular processes essential for tumor progression. In summary, this study demonstrated the efficacy of maceration as an optimal extraction method and confirmed the anticancer potential of cynthichlorine, particularly against glioblastoma. However, its efficacy against other types of cancers still requires further investigation.

The results of this study are in line with previous research on compounds derived from sea squirts, which have shown significant anticancer potential. Previous work on extracts of sea squirts, including *Ciona intestinalis*, has demonstrated antiproliferative and proapoptotic effects [32]. However, taxonomic errors have sometimes led to confusion in species identification, as was the case for some studies where *Ciona robusta* was used instead of *Ciona intestinalis* [33]. This type of confusion can compromise the reproducibility of the results and their correct interpretation. Meanwhile, Ecteinascidin-743 (ET-743), a compound extracted from *Ecteinascidia turbinata*, has shown promising results against soft tissue sarcoma and ovarian cancer, leading to its FDA approval. This compound is now used in hospitals worldwide, although variable clinical results and a re-evaluated toxicity profile have been observed [34]. However, this agent has been limited by mixed clinical results and a re-evaluated toxicity profile, highlighting the need for continued research to better understand the side effects of marine agents and refine their clinical use. In this context, cynthichlorine extracted from *Cynthia savignyi* appears to offer unique features, such as its high electronic stability and specific interactions with well-defined molecular targets, such as NM23-H2, which distinguish it from other compounds studied so far. This suggests that cynthichlorine could represent a promising potential therapeutic avenue for the treatment of certain cancers, particularly glioblastoma, but requires further research to evaluate its efficacy and safety on a larger scale.

## 4. Material and Methods

### 4.1. Marin Material

The tunicate *Cynthia savignyi* was collected in the Gulf of Tadjoura, located in the Tadjoura province of Djibouti (GPS coordinates: 11°45′23.7″ N, 42°53′39.8″ E), during an expedition conducted in April 2024. Sampling was carried out using an approach combining scuba diving and benthic dredging, thus allowing for the exploration of different depth zones between 0 and 40 m. In order to ensure traceability and rigorous identification, a control specimen (referenced under the number 2024CS5) was deposited and preserved at the Medicinal Research Institute, attached to the Center for Studies and Research of Djibouti (CERD).

### 4.2. Extraction and Isolation of Cynthichlorine

The extraction of cynthichlorine was performed using two distinct methods to optimize the yield and ensure the efficient separation of the metabolites of interest. In the first method, a maceration was performed by immersing the specimens previously cut into small pieces in a mixture of chloroform and ethanol (CHCl_3_/EtOH, 5:5, *v*/*v*) [35]. After a prolonged extraction period (12 h), the extract obtained was subjected to a fractional liquid–liquid extraction with solvents of increasing polarity, allowing for a selective separation of the compounds according to their chemical affinity. A first extraction with hexane allowed for the removal of lipids and highly apolar compounds, while a second extraction with ether led to the isolation of the compounds of interest, notably cynthichlorine. In the second method, a Soxhlet extractor was used to optimize the extraction of the active compounds [36]. The crushed specimens were subjected to successive extractions with solvents of increasing polarity, following the same protocol as the maceration (12 h for each cycle).

Once the extractions were completed using both methods, the selected ether extracts were filtered to remove impurities and then concentrated under reduced pressure using a rotary evaporator. This step allowed us to remove residual solvents and concentrate the extracted compounds for the subsequent purification steps.

The fractionation of the ether extracts obtained by both methods was carried out by atmospheric column chromatography, using silica gel as the stationary phase. The elution was carried out with an ether/methanol mixture (80:20, *v*/*v*), leading to the separation of several fractions. Among these, a yellow fraction was identified as being enriched in cynthichlorine.

The final purification of cynthichlorine was carried out by high-performance liquid chromatography (HPLC) in semi-preparative mode. The analysis was carried out on a reverse column, with elution performed using a mixture of acetonitrile/water (60:40, *v*/*v*). Using this technique, cynthichlorine was isolated in a highly purified form. The identity and structure of the compound were confirmed by various spectroscopic analyses [16].

### 4.3. Analytical Techniques

Chromatographic and spectroscopic analyses were performed to characterize cynthichlorine and ensure their structural identification.

The purification and chromatographic separation steps were carried out by combining different techniques [37]. Column chromatography was performed using Merck silica gel 60 (0.063–0.200 mm) under atmospheric pressure, ensuring the efficient separation of the fractions. For finer separation and advanced purification, high-performance liquid chromatography (HPLC) was performed with an Agilent 1260 Infinity II HPLC system. This system is equipped with a quaternary pump, offering a maximum pressure of 600 bar and a flow rate of up to 10 mL/min. It is coupled to a photodiode array (PDA) detector, allowing the precise spectral analysis of the purified fractions. Compatibility with semi-preparative columns such as the Luna 100 Å 10 µm (10 × 250 mm) allowed for the production of high-purity compounds. The integrated autosampler optimized the reproducibility of injections and improved the efficiency of the analytical process. In addition, thin-layer chromatography (TLC) was used as a preliminary analytical method. Separations were performed on preparative thin-layer chromatography (TLC) plates coated with Merck Kieselgel 60 PF254 silica gel containing gypsum, with a layer thickness of 1 or 2 mm. This technique facilitated the monitoring of purifications and the optimization of chromatographic conditions. All solvents used in these analyses were of HPLC grade and were used without modification to ensure the maximum reproducibility and precision of the results obtained. UV-VIS spectra were recorded in methanol using a Shimadzu UV-1601 spectrophotometer, allowing us to determine the characteristic absorption profiles of the molecules present. Infrared (FTIR) analysis was performed in the range of 1000 to 400 cm^−1^ using a Bruker Vertex 70 FTIR spectrophotometer, using the potassium bromide (KBr) pellet method to characterize the functional groups of the isolated compounds. The elucidation of the molecular structure was confirmed by nuclear magnetic resonance (NMR) using a Bruker Avance DPX-300 and Avance III 600 spectrometer. ^1^H and ^13^C NMR spectra were recorded in CDCl_3_ and C_3_D_6_O, respectively, applying the standard pulse programs provided by the manufacturer, allowing for a detailed analysis of the chemical environments of protons and carbons. High-resolution mass spectrometry (HRMS) was performed on a Bruker BioApex 47 FT mass spectrometer, equipped with an electrospray ionization (ESI) source, in positive ion detection mode, to accurately determine the molecular mass of the isolated compounds [38,39].

Cynthichlorine: pale yellow solid. UV (MeOH) λ_max_ (log ε) 225 (4.27), 263 (3.88), 315 (2.94) nm. IR (CHCl_3_) ν cm^−1^: 3435 (O–H, s), 3205 (N–H, m), 3110 (=C–H, w), 2950 (>C–H, s), 2850 (C–H, m), 1765 (C=O, s), 1745 (C=O, m), 1620 (C=C, s), 1590 (C=C, m). ^1^H NMR (CDCl_3_, 300 MHz) δ 10.70 (br s, NH), 8.24 (br s, H-1), 7.22 (d, J = 2 Hz, H-4), 7.26 (d, J = 2 Hz, H-6), 3.45 (d, J = 17 Hz, H-8a), 3.71 (d, J = 17 Hz, H-8b), 3.57 (s, H-10). ^13^C NMR (C_3_D_6_O, 75 MHz) δ 173.6 (s, C-2), 168.6 (s, C-9), 137.8 (s, C-7a), 131.8 (s, C-3a), 130.5 (d, C-6), 129.0 (s, C-5), 123.3 (d, C-4), 116.4 (s, C-7), 60.4 (s, C-3), 52.6 (q, C-10), 42.7 (t, C-8). HREIMS *m*/*z* 306.9594 [M+], calcd. for C_11_H_8_NO_3_Cl_3_: 306.9570. EI-MS *m*/*z* (rel. int.): 311 (18), 309 (58), 307 (59), 272 (16), 264 (29), 262 (24), 250 (26), 248 (28), 232 (55), 230 (100), 215 (60), 213 (98), 187 (50), 185 (79), 152 (22), 150 (67), 125 (29), 123 (87).

### 4.4. In Vitro Cytotoxicity Assay

The cytotoxic activity of cynthichlorine was assessed using the CellTiter-Glo assay, a luminescence-based method for measuring cell viability. Different concentrations of cynthichlorine (0.5, 1, 5, 10, 20, and 50 μg/mL) were prepared. Five human cancer cell lines (U87-MG, U2OS, NCI-N87, HCT116, and A2780) were obtained from reference collections such as (ECACC, Porton Down, UK), the American Type Culture Collection (ATCC, Rockville, MD, USA), and the German Collection of Microorganisms and Cell Cultures of the Leibniz Institute (DSMZ, Braunschweig, Germany). Cells were cultured in specific media (DMEM or RPMI-1640) supplemented with fetal bovine serum (10% FBS), glutamine (1%), and penicillin/streptomycin (1%) and incubated at 37 °C under an atmosphere containing 5% CO_2_. Daily monitoring was performed under an inverted microscope to monitor their morphology and detect possible contamination. Aliquots of 100 μL of the cell suspension (5 × 10^3^ cells/well) were seeded in 96-well plates and incubated for 24 h to promote their adhesion. Subsequently, cells were exposed to cynthichlorine and incubated for 72 h. After treatment, 100 μL of CellTiter-Glo reagent was added, followed by incubation for 15 min at room temperature in the dark. Luminescence was measured by spectrophotometry, allowing cell viability to be determined. IC_50_ values were calculated from a nonlinear dose–response curve. As positive controls, the following reference anticancer agents were used: temozolomide (U87-MG), cisplatin (U2OS, NCI-N87), oxaliplatin (HCT116), and paclitaxel (A2780). The ethical approval of the study was validated by the ethics committee of ESTK-USMS (Morocco), and the genetic data were archived under number 2024-0901-0001 [40,41].

### 4.5. Computational Analysis

Computational chemistry investigations were integrated into this study in order to deepen the theoretical analysis of the structural and electronic properties of cynthichlorine, a molecule of interest, as well as its interaction with the major oncogenic proteins involved in tumorigenesis. Two complementary approaches were implemented, combining the modeling of the molecular interactions of cynthichlorine by determining specific energy descriptors and a molecular docking study aimed at characterizing the modes of interaction with target proteins.

In the first approach, the Merck Molecular Force Field 94 (MMFF94) method was adopted to model the inter-atomic interactions within the molecule in molecular mechanics [42]. Several electronic descriptors were extracted to characterize the reactive properties of cynthichlorine by ChemBioDraw (13.0), including the energy of the frontier molecular orbitals, occupied (E_HOMO_) and unoccupied (E_LUMO_), as well as the energy gap (E_GAP_), a key parameter influencing chemical reactivity and electronic stability. Other quantum descriptors, such as chemical hardness, electronegativity, the global electrophilicity index, and molecular flexibility, were calculated to obtain a more detailed characterization of the electronic and structural properties of the molecule.

The second computational approach is based on a molecular docking study aimed at elucidating the interaction mechanisms between cynthichlorine and three proteins (ATAD2, NM23-H2, and INTS3/SAGE1) that are involved in the regulation of transcription and chromatin structure, which has implications in cancer [43]. The three-dimensional structures of these proteins were extracted from the Protein Data Bank (PDB) database under the codes 8JUY, 3BBB, and 8HPP. Prior to docking, protein structure optimization was performed using Swiss-Pdb Viewer v4.1 software, followed by the rigorous preparation of molecular receptors, including the removal of water molecules and heteroatoms, the addition of polar hydrogens, and partial charge assignment using the Kollman method. Cynthichlorine was saved in a .pdb format, and its geometry was optimized by Density Functional Theory (DFT) calculations using the B3LYP/6-311G formalism. Molecular docking was performed using AutoDock Vina 1.1.2 software, with a 40 Å exploration grid in the three directions x, y, and z to map potential binding sites (Table 10). Ligand–receptor interaction analysis was performed using BIOVIA Discovery Studio software (Studio, 2021), allowing for the identification and characterization of key interactions, such as hydrogen bonds, hydrophobic interactions, and metal coordinations, involved in the stabilization of the complexes formed. This structure–function analysis provides valuable insights into the pharmacological potential of cynthichlorine as a bioactive agent targeting oncogenic proteins.

### 4.6. Statistical Approach

Numerical data were collected from three replicates for each test to ensure the accuracy of the results. A type A uncertainty assessment was performed to statistically analyze the data obtained. To assess the significance of the tests, statistical analysis was conducted using Student’s *t*-test with a significance level set at *p* < 0.05 [44].

The analysis of differences between samples was performed by applying Student’s *t*-test, a commonly used method to compare two means. This test is based on the assumption that the errors follow a normal distribution and that the standard deviations of the samples are homogeneous. The means and standard deviations were calculated, and then an experimental t-value was determined and compared to the theoretical critical value. When the experimental value exceeds the critical value, the null hypothesis is rejected, indicating a significant difference between the samples [45].

A Box–Behnken experimental design was used to optimize the experimental parameters by testing several factors simultaneously. This methodology allowed us to structure the experimental tests while limiting the number of tests required. By applying this model, the effects of interactions between factors were studied in order to identify their influence on the measured responses. This design was preferred because of its efficiency in evaluating quadratic effects without requiring an excessive number of experiments [18].

A principal component analysis (PCA) was performed in order to reduce the dimensionality of correlated variables and to extract uncorrelated principal components. This approach was applied to analyze the correlation between the different parameters, particularly in the study of cytotoxic properties and parameters resulting from computational analysis. The application of PCA made it possible to identify influential factors by grouping highly correlated variables in an optimized projection space [46].

All statistical analyses, experimental modeling, and graphical representations were carried out using Excel 2010, XLSTAT (version 2016) and Design-Expert 13 software. These tools made it possible to optimize the experimental studies by facilitating the interpretation of results and the adjustment of statistical models.

## 5. Conclusions

The present study aimed to evaluate and optimize the extraction method of cynthichlorine from the ascidian *Cynthia savignyi* while exploring its potential as a cytotoxic agent against various cancer lines. The results revealed that the maceration method offered a significantly higher extraction yield than Soxhlet extraction due to the thermal sensitivity of the molecule. An optimization study showed that extraction temperature and time negatively influenced the yield, suggesting that low temperature and short time conditions are ideal for maximizing extraction. Regarding cytotoxic activity, cynthichlorine demonstrated the significant inhibition of cell growth, especially against the U87-MG (glioblastoma) cell line, even surpassing some reference chemotherapeutic agents such as cisplatin and temozolomide. Computational analysis provided additional insights into the electronic stability of the molecule, suggesting that it exhibits low chemical reactivity and high stability in a physiological environment. Molecular docking highlighted NM23-H2 and ATAD2 proteins as potential targets, with a higher binding affinity for the former, paving the way for future research on cynthichlorine as a targeted therapy against certain cancers. In conclusion, this study proposes cynthichlorine as a promising candidate for the development of novel anticancer therapeutic strategies while highlighting the need for further research on its mechanisms of action and biological interactions.

## Figures and Tables

**Table 1:** Results of the comparison of cynthichlorine yields between maceration and Soxhlet methods using Student’s *t*-test.

Parameter	Maceration	Soxhlet	Calculated Value
Sample Size	n_1_ = 11	n_2_ = 11	-
Mean	2.2	1.0	-
Standard Deviation	s_1_ = 0.2	s_2_ = 0.2	-
Degrees of Freedom	-	-	ν = 20
Pooled Standard Deviation	-	-	s * = 0.2
Test Statistic	-	-	t_exp_ = 16.3
Critical t-Value	-	-	tth = 2.1
Test Decision	-	-	Reject H_0_ (Significant Difference)

**Table 2:** Factor levels influencing maceration extraction.

Level	Temperature (°C)	Time (h)	Alcohol (%)
−1	25	12	50
0	30	18	75
+1	35	24	100

**Table 3:** Experimental design and obtained yields.

Trial	Temperature (°C)	Time (h)	Alcohol (%)	Yield (%)
1	25	12	75	3.1
2	35	12	75	2.2
3	25	24	75	2.1
4	35	24	75	1.7
5	25	18	50	2.4
6	35	18	50	2.1
7	25	18	100	2.6
8	35	18	100	2.0
9	30	12	50	2.3
10	30	24	50	2.2
11	30	12	100	2.1
12	30	24	100	1.9
13	30	18	75	2.1

**Table 4:** Estimation of factor coefficients.

Factor	Coefficient Estimate	Standard Error	VIF
Mean Yield	2.2	0.1	-
Temperature (A)	−0.3	0.1	1.0
Time (B)	−0.2	0.1	1.0
Alcohol (C)	−0.1	0.1	1.0
Interaction AB	0.1	0.1	1.0
Interaction AC	−0.1	0.1	1.0
Interaction BC	−0.1	0.1	1.0

**Table 5:** Analysis of Variance (ANOVA) for the reduced cubic model.

Effect	Sum of Squares	Df	Mean Square	f-Value	*p*-Value
Model	1.1	6	0.18	3.50	0.07
Temperature	0.60	1	0.60	11.36	0.01
Time	0.40	1	0.40	7.61	0.03
Alcohol	0.02	1	0.02	0.37	0.56
Interaction Temperature:Time	0.06	1	0.06	1.17	0.32
Interaction Temperature:Alcohol	0.02	1	0.02	0.42	0.53
Interaction Time:Alcohol	0.00	1	0.00	0.04	0.83
Residual	0.31	6	0.05	-	-
Total	1.44	12	-	-	-

**Table 6:** Model fit statistics.

Parameter	Value
Residual Standard Deviation	0.2
R^2^	0.78
Adjusted R^2^	0.56
Coefficient of Variation (%)	10.41
Predicted R^2^	−0.20
Adequate Precision	6.05

**Table 7:** IC_50_ (µg/mL) values of cynthichlorine and other anticancer agents for cytotoxicity assessment against U87-MG, U2OS, NCI-N87, HCT116, and A2780 cell lines.

Compounds	U87-MG	U2OS	NCI-N87	HCT116	A2780
Cynthichlorine	0.162 ± 0.020	0.373 ± 0.040	0.576 ± 0.060	0.505 ± 0.055	0.529 ± 0.055
Cisplatin	0.185 ± 0.025	0.002 ± 0.001	0.004 ± 0.001	0.455 ± 0.060	0.788 ± 0.085
Temozolomide	0.003 ± 0.001	0.840 ± 0.095	0.795 ± 0.085	0.889 ± 0.095	0.811 ± 0.095
Oxaliplatin	0.387 ± 0.045	0.421 ± 0.045	0.344 ± 0.040	0.002 ± 0.001	0.293 ± 0.095
Paclitaxel	0.235 ± 0.030	0.166 ± 0.020	0.358 ± 0.040	0.255 ± 0.030	0.003 ± 0.001

**Table 8:** Quantum descriptors of cynthichlorine.

Descriptors	Symbol	Equation	Numerical Value
Energy of the lowest unoccupied molecular orbital	E_LUMO_	-	−0.619 eV
Energy of the highest occupied molecular orbital	E_HOMO_	-	−9.441 eV
Energy gap	E_GAP_	$E_{LUMO}-E_{HOMO}$	8.822 eV
Chemical hardness	η	$\frac{E_{LUMO}-E_{HOMO}}{2}$	4.411 eV
Electronegativity	χ	$-\left(\frac{E_{LUMO}+E_{HOMO}}{2}\right)$	5.030 eV
Electrophilicity index	ω	$\frac{\chi^{2}}{2\eta }$	2.868 eV
Molecular flexibility	S	$\frac{1}{2\eta }$	0.113 eV^−1^

**Table 9:** Energetic data of molecular docking interactions between cynthichlorine and proteins 8JUY, 3BBB, and 8HPP.

Proteins	Binding Affinity (kcal.mol^−1^)	p*Ki*	Ligand Efficiency (kcal.mol^−1^)
8JUY	−6.5	4.77	0.3611
3BBB	−7.0	5.13	0.3889
8HPP	−5.4	3.96	0.3000

**Table 10:** Active site coordinates of three proteins.

Parameters	8JUY	3BBB	8HPP
Coordinates	x = 168.430 Å	x = −0.142 Å	x = −32.792 Å
	y = 152.569 Å	y = 0.219 Å	y = −49.105 Å
	z = 156.243 Å	z = 43.267 Å	z = 6.061 Å
Dimensions	x = 144 Å	x = 76 Å	x = 104 Å
	y = 161 Å	y = 93 Å	y = 90 Å
	z = 142 Å	z = 86 Å	z = 118 Å

## Data Availability

Data are contained within the article.
